# Journal statistics, coping strategy with upcoming scholarly journal publishing environment including Plan-S, and appreciation for reviewers and volunteers

**DOI:** 10.3352/jeehp.2019.16.41

**Published:** 2019-12-28

**Authors:** Sun Huh

**Affiliations:** Department of Parasitology and Institute of Medical Education, Hallym University College of Medicine, Chuncheon, Korea; The Catholic University, Korea

## Being indexed in 2 literature databases

This year has brought us some good news for the *Journal of Educational Evaluation for Health Professions* (JEEHP):

First, JEEHP was accepted for inclusion in Scopus by Scopus Content Selection and Advisory Board (CSAB) [1]. Although JEEHP has been indexed in Scopus since 2016 as a MEDLINE-source journal, it had to be re-evaluated by CSAB. The journal had added merits of having references of articles and a directory of the countries of submitting authors in a separate field, leading to more fruitful bibliometric findings of the journal.

Second, JEEHP was included in Korea Citation Index (KCI) in April this year, an abstract and reference database maintained by the National Research Foundation of Korea (NRF), which is certainly a prestigious achievement because NRF had not allowed online-only journal without regular frequency to be included in KCI thus far. It had only allowed Scopus, SCIE (Science Citation Index Expanded), SSCI (Social Science Citation Index), or A&HCI (Arts and Humanities Citation index)-indexed journals to be included in KCI even if the journal has no frequency. JEEHP is believed to be the first scholarly journal in Korea published as online-only without regular frequency since 2006 [2], providing a good chance to be cited by scholarly educational journals in Korea.

## Adoption of new technology in journal web sites

**Fig. 1.**
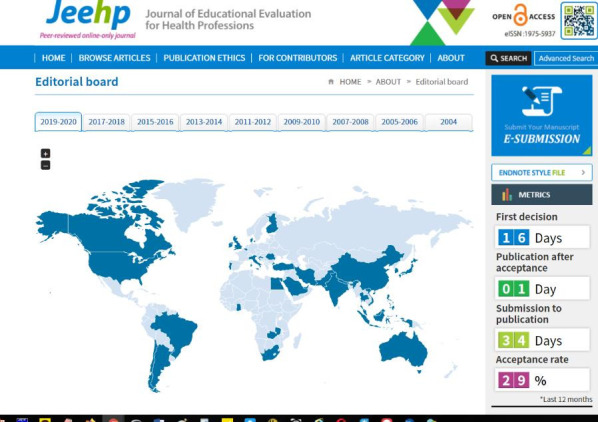
Screenshot of countries of editorial board members and review process counter adopted in 2019 to *Journal of Educational Evaluation for Health Professions*. Available from: https://www.jeehp.org/.

New technology was adopted by the JEEHP this year: (1) review process counter from October 16; (2) country map of authors in each year with a table of content and editorial board members from December 9; (3) country flag of the authors and the editorial board members from December 9; and (4) distribution of countries of the authors in metrics from December 9. Among these, review process counter can be a good incentive to submitting authors. Authors want decisions on their manuscripts as soon as possible, and currently the median value of the period to the first decision is 16 days (Fig. 1). Countries of the submitting authors and the editorial board members are also important to show the international diversity of the journal, and flags and maps will contribute to providing an easy and quick grasp of this international diversity.

## Bibliometric analysis

**Fig. 2.**
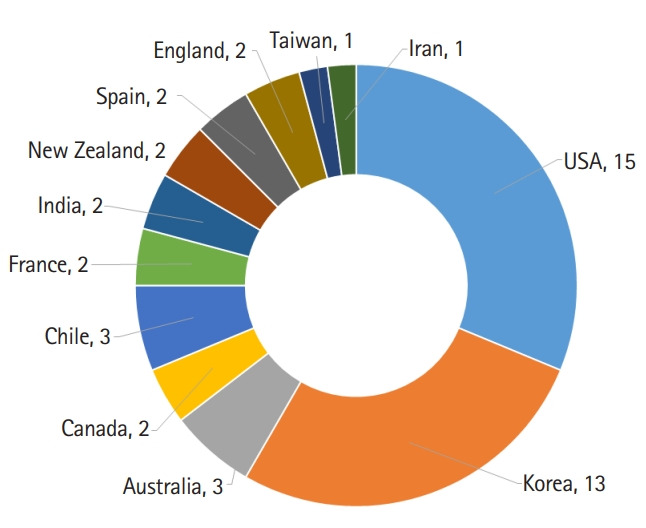
Countries of authors of *Journal of Educational Evaluation for Health Professions* in 2019.

**Fig. 3.**
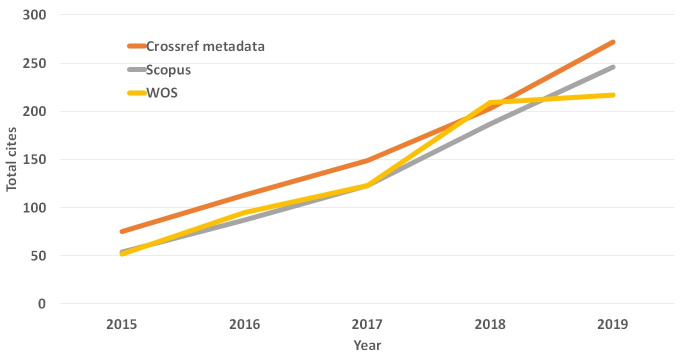
Total cites of *Journal of Educational Evaluation for Health Professions* in CrossRef metadata, Scopus, and Web of Science Core Collection (WOS) from 2015 to 2019.

**Fig. 4.**
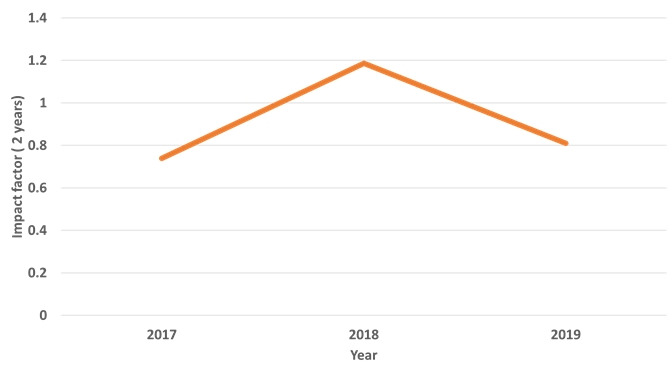
Two-year impact factor of *Journal of Educational Evaluation for Health Professions* calculated in Web of Science Core Collection from 2017 to 2019.

Journal performance can be presented based on the bibliometric indexes including total citations and an impact factor. Twelve countries of the authors of 41 articles published this year are presented in Fig. 2. The United States of America was ranking top with 15 articles out of 41. Total citations from Scopus, Web of Science Core Collection, and CrossRef meta-database are presented in Fig. 3, showing a trend of continuous increase.. The 2 year-impact factors in Web of Science for recent years are calculated and presented in Fig. 4. There was a decrease in impact factor in 2019, which may be attributed to rare citation in literature on licensing examination. Nine articles on licensing examination published in 2017 were cited 10 times, while 5 articles in 2018 were cited 3 times up to now, which is lower than the citation frequency of other articles. Because articles on health licensing examination are rare worldwide, it is also difficult to be cited frequently. However, it may serve to be an evidence of this journal’s uniqueness that is devoted to publishing articles on licensing examination.

## Basic statistics on submission and review

The fate of manuscripts submitted to this journal up to December 27, 2019 is summarized in Table 1.

## Change in publishing environment outside Korea

It is anticipated that the enactment of immediate open access publication without embargo period for articles will soon be supported by the US federal funding agencies including National Science Foundation and National Institute of Health [3,4]. It may be an extension of the public access policy by the above 2 funding institutes, which mandates free access after 1-year embargo period if the articles are supported by these funding agencies. It is a fortifying policy for open access publication. It may be a good chance for the journal to receive research results that had received US federal funding, because it is the diamond or platinum open access one without embargo period nor article processing charge. However, the situation in Europe is not favorable, where “all scholarly publications on the results from research funded by public or private grants provided by national, regional and international research councils and funding bodies, must be published in open access journals without embargo from 2021” according to Plan-S [5]. There are basic, mandatory, and recommended requirements to be eligible to receive the manuscripts supported by European funding bodies. Out of them, one basic requirement of “copyright owned by authors or institutes” cannot be fulfilled by the journal, because this journal is owned by the public institute publisher and all publishing cost is supported by the publisher. This year, 20% of the published articles were from Europe, although most of those articles were not supported by research grants. The JEEHP should be prepared for the situation in which manuscripts funded by European funding agencies cannot be accepted. However, at present, there seems to be no way to overcome this obstacle, and this may apply to other public or non-profit organization journals as well. I just anticipate a change in the principle of Plan-S on the ownership of copyright. There is no problem in publishing the journal as open access without embargo nor article processing charge although the copyright is owned by the publisher in Korea. Furthermore, the open access policy which may be enacted by the Korean Government in near future should be followed-up and discussed to evade the situation in Europe like Plan-S principle of copyright ownership.

## Appreciation for reviewers and volunteers

I am deeply indebted to following reviewers. Without their tremendous help, I couldn’t have edited and published the journal so beautifully:

Abdolghani Abdollahimohammad, Neeka Akhavan, Malhotra Anita, Marcela Andrea Antúnez-Riveros, Colin Block, Raul Alfredo Borracci, Megan Brown, Su-Jin Chae, Lucia Chehade, A Ra Cho, Kyunghee Chun, Cheol-Woon Chung, Derek Clewley, Fabrizio Consorti, Mariana D'amico, Saeideh Daryazadeh, Olivos David, Upreet Dhaliwal, Ilse Erich, Christian Ezeala, Min Huang, Karen Huhn, Yera Hur, Geum-Hee Jeong, Williams Joy, Im Jung Jun, Taehoon Kang, Chien Kevin, Ahmed Khalifa Khalif, Kyung Won Kim, Seock-Ho kim, Young Min Kim, Ju-Yeun Lee, Young Hwan Lee, Eun Young Lim, Paula Kay Martin, argetto-Fernandez Miguel, Kyung-A Nam, Younjae Oh, Robin Parish, Bhina Patria, Rano Mal Piryani, Agarwal Ramesh, Marcus Roll, Dong Gi Seo, Jihyun Seo, Ravi Shankar, Eunah Shin, Mohan Sunkad, Chloe Thabet, Gideon Victor, Yanhua Yi, and Hon Yuen.

Voluntary work of audio recording of the abstract when authors were not available, was done by Tom Huh, a student with a major in Biology, Korea University, Seoul, Korea.

I am very happy to have published 41 articles this year, including 30 research articles, 2 reviews, 3 brief reports, 1 technical report, 3 editorials, 1 educational/faculty development materials, and 1 announcement. Next year is the year of “white rat” according to the sexagenary cycle. Rats stand for multi-parity, diligence, and abundance of riches in Korean culture. I hope the journal to also prosper in receiving an adequate number of interesting and practical manuscripts for medical health educators. I will be more than happy if the contents of this journal are adopted by many experts in medical health educational fields worldwide.

## Figures and Tables

**Table 1.** Basic statistics on the fate of the submitted manuscripts to the *Journal of Educational Evaluation for Health Professions* in 2019

	Number	Content
Manuscripts submitted	147	One article (DOI: https://doi.org/10.3352/jeehp.2019.16.4) was submitted in 2018; however, it was re-submitted after new formatting. Therefore, it was included in 2019
Manuscripts determined without review	92	Unsuitable, 90; other reasons, 2;
Manuscripts reviewed and determined	49	Accepted and published, 40; rejected 8; withdrawal, 1
Manuscripts under review or revision	6	Under revision, 3; under review, 3
Acceptance rate (%)	28.4	40/141=0.284
Median time from submission to the first decision (day)	16	-
Median time from submission to publication (day)	34	-
